# Development and Validation of an m6A-Derived Prognostic Signature in Lung Adenocarcinoma

**DOI:** 10.7150/jca.134792

**Published:** 2026-05-29

**Authors:** Zheng Shao, YongLi Situ, Bairu Lai, Ying Xu, Jv Chen, Li Deng, QinYing Xu, Meng Liang, Zhu Liang, ZhanQiang Xie

**Affiliations:** 1 School of Basic Medical Sciences, Guangdong Medical University, Zhanjiang, Guangdong, 524023, China.; 2 School of Medical Technology, Guangdong Medical University, Dongguan, Guangdong, 523808, China.; 3 Department of Pharmacy, Affiliated Hospital of Guangdong Medical University, Zhanjiang, Guangdong, 524023, China.; 4 Department of Thoracic Surgery, Affiliated Hospital of Guangdong Medical University, Zhanjiang, Guangdong, 524023, China.

**Keywords:** lung adenocarcinoma, m6A modification, prognostic model, tumor immune landscape, treatment response prediction

## Abstract

**Background:**

Lung adenocarcinoma (LUAD) exhibits extensive molecular heterogeneity and poor prognosis, necessitating novel epigenetic biomarkers. N6-methyladenosine (m6A) modification, a pivotal epigenetic regulator of RNA, is frequently dysregulated in LUAD, yet its systematic roles in clinical prognosis and the tumor microenvironment (TME) remain poorly elucidated.

**Methods:**

Transcriptomic profiles and clinical data from large-scale cohorts were integrated and analyzed. Unsupervised consensus clustering based on 47 m6A-related genes (MRGs) was performed to distinguish distinct molecular subtypes. A prognostic model was developed via LASSO-Cox regression algorithm and further validated in multiple independent cohorts. Immune infiltration, tumor mutational burden (TMB), immunotherapy response (TIDE/IPS), and chemotherapy sensitivity were analyzed, complemented by single-cell RNA sequencing.

**Results:**

Two distinct m6A-related gene clusters (mRGclusters A and B) were identified. Patients in cluster B exhibited inferior survival outcomes, higher m6A pathway activity, and significant enrichment of cell cycle-related pathways. The eight-gene signature successfully stratified patients into high-risk (HR) and low-risk (LR) subgroups. Patients in the HR group presented significantly worse overall survival (P < 0.05), higher TMB levels, and upregulated expression of multiple immune checkpoint genes such as *LAG3* and *PDCD1*. *CSMD3* mutations were capable of improving the survival of HR patients by facilitating the infiltration of natural killer cells and follicular helper T cells. The signature independently predicted prognosis (AUC: 0.70-0.84) and treatment response: LR patients favored immunotherapy (lower TIDE, higher IPS), while HR patients were sensitive to chemotherapy (e.g., Bosutinib, Tozasertib).

**Conclusion:**

This transcriptome-derived m6A-associated prognostic model can effectively predict clinical survival outcomes and therapeutic response in LUAD patients. Combined with immune landscape, genomic mutation profiles and single-cell transcriptomic evidence, this signature provides a reliable basis for personalized risk stratification and rational treatment choice.

## Introduction

LUAD, the most prevalent subtype of lung cancer (accounting for ~40% of cases), is characterized by early metastatic potential and extensive molecular heterogeneity, severely hindering accurate prognosis assessment and personalized treatment 1-3. Despite advances in targeted therapies (e.g., EGFR inhibitors) and immune checkpoint blockade, the 5-year overall survival (OS) rate of LUAD patients remains below 20% 4, highlighting an urgent need to identify novel molecular biomarkers for improved risk stratification and therapeutic guidance.

N6-methyladenosine (m6A), the most abundant internal epigenetic modification in eukaryotic mRNA, regulates essential biological processes including RNA stability, translation efficiency, and alternative splicing 5,6. Dysregulation of m6A-related genes (MRGs) contributes to LUAD tumorigenesis and progression: for example, METTL3 overexpression promotes LUAD cell growth, while FTO downregulation correlates with poor patient outcomes 7-10. However, current research primarily focuses on single-gene validation, lacking systematic analyses of MRG expression profiles, clinical associations, immune microenvironment (TME) interactions, and genomic alterations. Additionally, m6A-based prognostic models constructed from bulk transcriptomic data, further interpreted with immune, mutational, and single-cell profiles to predict patient outcomes and treatment responses, still require comprehensive development and validation.

To address these gaps, this study aimed to construct a novel m6A-related prognostic signature for LUAD using transcriptomic and clinical data from large cohorts. Specifically, we sought to: (1) identify m6A-modified molecular subtypes via unsupervised clustering of MRGs; (2) develop and validate a prognostic model based on key MRGs using Cox regression and LASSO algorithms; (3) explore correlations between m6A profiles, immune cell infiltration, and tumor mutational burden (TMB); and (4) predict therapeutic vulnerabilities associated with m6A signatures. These analyses aim to clarify the role of m6A modification in LUAD progression and facilitate the translation of epigenetic markers into clinical practice for precision oncology.

## Materials and Methods

### Data Acquisition and Preprocessing

Transcriptomic and clinical data for TCGA-LUAD patients were obtained from the UCSC Xena portal (https://xenabrowser.net/datapages/). Independent validation cohorts (GSE41271, 182 samples; GSE42127, 133 samples) were sourced from the Gene Expression Omnibus (GEO) database (https://ncbi.nlm.nih.gov/geo/). Single-cell RNA sequencing data from GSE149655 (GEO) were also included. Patients with matching RNA sequencing data and survival information were retained, with duplicate records removed, resulting in 500, 182, and 133 LUAD samples for TCGA-LUAD, GSE41271, and GSE42127, respectively. The hallmark gene sets were downloaded from the Molecular Signatures Database (MSIGDB).

### MRGs Selection

MRGs were identified through the GeneCards database (https://www.genecards.org/) by searching with the keyword "m6A". In GeneCards, the relevance score is a composite indicator that evaluates the strength of association between a gene and the query term, and a threshold of ≥ 2.0 was adopted to ensure reliable biological relevance. After filtering for protein-coding genes, 47 MRGs were included. Although several published studies and reviews have summarized core m6A regulatory gene sets (writers, erasers, readers), we used a broad GeneCards-based strategy to include both core regulators and genes indirectly associated with m6A functions, allowing a more comprehensive exploration of m6A-related expression patterns in LUAD. This gene set therefore represents a broad m6A-associated gene panel rather than a strictly defined core m6A regulatory complex. The full list of MRGs is provided in Supplementary Table S1. The expression matrix of MRGs in LUAD samples was extracted and normalized for subsequent analysis.

### Unsupervised Consensus Clustering

Unsupervised consensus clustering was performed using the R package ConsensusClusterPlus (v1.66.0) to classify LUAD patients into molecular subtypes based on MRG expression. The optimal number of clusters was determined by evaluating consensus scores across k = 2 to k = 9, with parameters set as: maxK = 9, reps = 50, pItem = 0.8. Patients were stratified into two clusters (mRGcluster A and B) for downstream analysis.

### Survival and Functional Enrichment Analysis

Kaplan-Meier (KM) survival curves were generated using the R package survival (v3.8-3) to compare OS between mRGclusters, with significance assessed by log-rank test. Single-sample Gene Set Enrichment Analysis (ssGSEA) was conducted using the R package GSVA (v1.50.5) to calculate m6A pathway activity scores for each cluster. Gene Set Enrichment Analysis (GSEA) was performed to compare m6A pathway activation between mRGcluster A and B, using the 47 MRGs as the custom gene set for GSEA analysis.

### Immune Infiltration Analysis

Immune cell infiltration levels were assessed using the ssGSEA and CIBERSORT methods. CIBERSORT was performed with 1000 permutations, and immune cell fractions with P < 0.05 were retained for subsequent analysis. The differential infiltration of immune cell subsets between clusters was compared using the Wilcoxon rank-sum test.

### Construction of m6A-Related Prognostic Model

Differentially expressed genes (DEGs) between mRGcluster A and B were identified using the R package limma (v3.58.1) with thresholds of |log2FC| ≥ 1 and *P* < 0.001. Univariate Cox regression analysis was applied to the DEGs to identify prognosis-associated genes (*P* < 0.001), followed by LASSO-penalized Cox regression (R package glmnet, v4.1-8) for dimensionality reduction and establishment of a prognostic model. LASSO Cox regression was implemented with 10-fold cross-validation, and the optimal lambda value was determined according to the minimum criterion. Patients were classified into high-risk (HR) and low-risk (LR) groups based on the median risk score.

### Model Validation and Prognostic Analysis

The optimal cutoff value for risk stratification was defined as the median risk score. Notably, the median value was independently calculated in the TCGA training cohort, GSE41271, and GSE42127, respectively, rather than being transferred directly from the training set to validation cohorts. This approach ensures that the cutoff adapts to data distribution in each cohort but may also lead to potential overestimation of predictive performance.

Survival differences between HR and LR groups were evaluated using KM analysis and log-rank test in both TCGA-LUAD and validation cohorts (GSE41271 and GSE42127). Time-dependent receiver operating characteristic (ROC) curves were generated to assess the model's predictive accuracy for 1-, 3-, and 5-year OS using the R package timeROC (v0.3). Expression patterns of all prognostic genes were visualized across risk groups, and their individual prognostic values were validated via KM analysis.

### Clinical Correlation and Nomogram Construction

Univariate and multivariate Cox regression analyses were performed to evaluate the independent prognostic value of the risk score, adjusting for clinical features (stage, age, gender, and *MKI67* expression) in TCGA-LUAD and validation cohorts. A clinical nomogram integrating the risk score and clinical parameters was constructed using the R package 'rms' (v6.7-1), with predictive performance assessed by calibration plots and time-dependent ROC curves. Calibration curves of the nomogram were validated using 10-fold cross-validation to evaluate predictive stability.

### Drug Sensitivity and Immunotherapy Response Prediction

The response to immunotherapy was predicted using the Tumor Immune Dysfunction and Exclusion (TIDE) algorithm (http://tide.dfci.harvard.edu/) and the Immunophenoscore (IPS, https://tcia.at/). A higher TIDE score indicates a greater risk of immune evasion and a weaker treatment response, while a higher IPS value reflects greater sensitivity to immune checkpoint inhibitors and enhanced responsiveness to immunotherapy. Chemotherapy drug sensitivity was assessed by predicting the half-maximal inhibitory concentration (IC50) values for 367 drugs, utilizing the ridge regression method implemented in the R package OncoPredict (v1.2), based on the Genomics of Drug Sensitivity in Cancer (GDSC1) dataset. The differences in sensitivity between HR and LR groups were analyzed using the Wilcoxon test.

### Single-Cell RNA Sequencing

ScRNA sequencing dataset GSE149655 contained two patients diagnosed with LUAD, and both tumor tissues and paired adjacent normal lung tissues were collected from each patient. The 10× sequencing data were imported and transformed into a Seurat object, followed by cell quality control filtering with the following thresholds: the number of detected genes per cell was set between 200 and 10,000, the total UMI count per cell was required to be no less than 1,000, and the proportions of mitochondrial and ribosomal gene counts were both limited to ≤ 0.2. After stringent filtering, a total of 7,685 high-quality cells were retained for subsequent downstream analyses. All filtered gene-barcode matrices across samples were integrated using Seurat v5.1.0 to correct and eliminate inter-sample batch effects, and clustering analysis was further performed with the number of principal components (PCs) set to 7 and resolution set to 1.0. Global dimensionality reduction was conducted via the UMAP algorithm. For auxiliary cell type annotation, the R package “singleR” (v2.4.1) was used together with marker genes from the CellMarker database for manual annotation of distinct clusters. Cell type annotation was performed on the integrated scRNA-seq dataset using canonical markers and transcriptomic clustering, with consistent cell labels retained when grouping cells by tissue origin (adjacent normal vs. tumor). Epithelial cells were identified by EPCAM and KRT19. Malignant epithelial cells (Malign_Epi) were separated from normal alveolar epithelial cells based on distinct clustering and LUAD-specific gene expression. The sporadic Malign_Epi cells in adjacent normal tissue likely reflect pre-neoplastic field-effect changes, rather than real tumor cell contamination. Finally, the expression and distribution of the 8 signature genes across different cell populations were investigated.

### Statistical Analysis

All statistical analyses were performed in R (v4.3.2). Continuous variables were compared using the Wilcoxon rank-sum test, and correlations were evaluated via Pearson's test. A two-tailed *P* < 0.05 was considered statistically significant.

## Results

### mRGs: Clustering, Prognosis, and Immune Infiltration in LUAD

**Figure 1:**
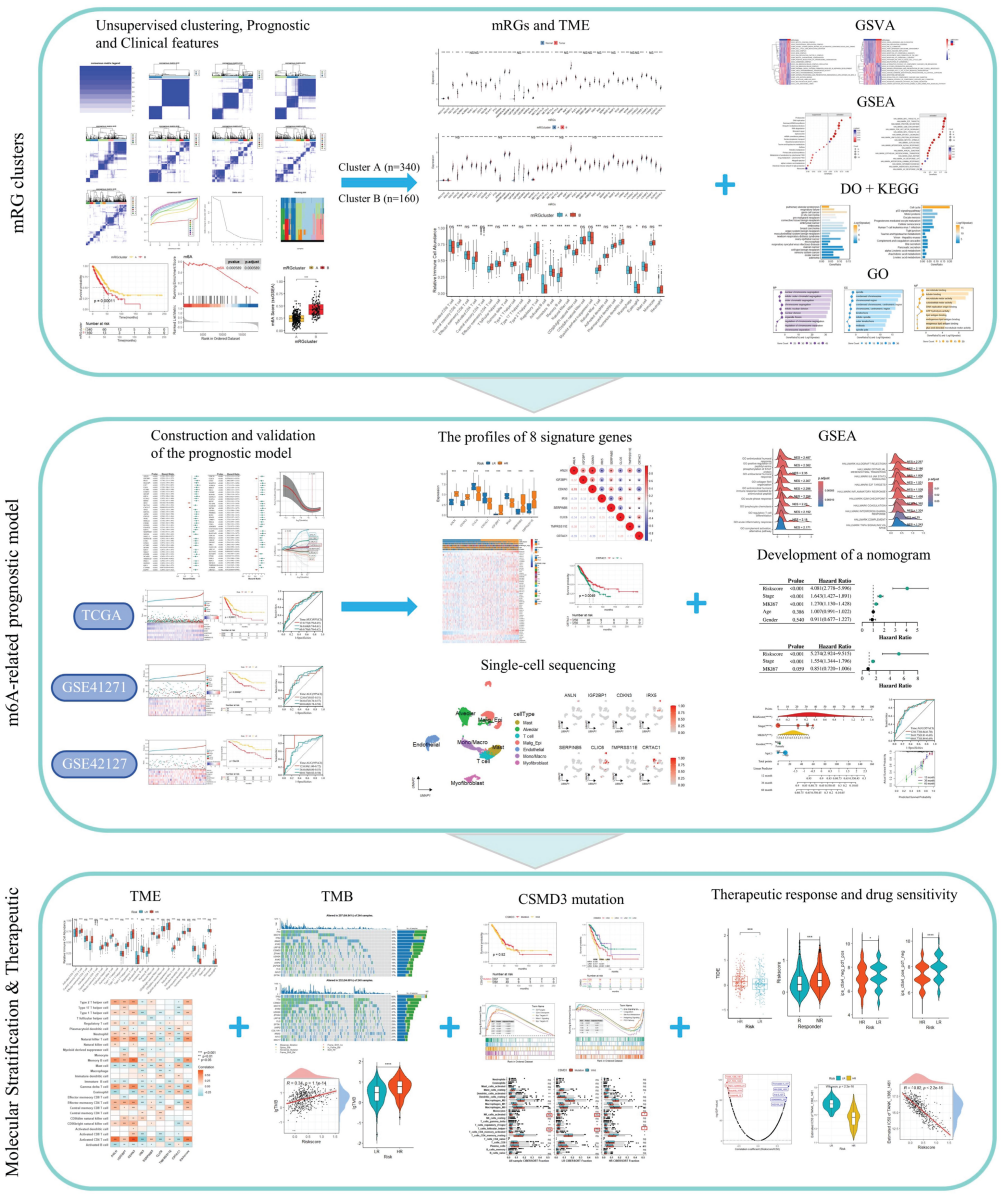
** Flow Chart of This Study.** *P < 0.05; **P < 0.01; ***P < 0.001; ns, not significant.

**Figure 2:**
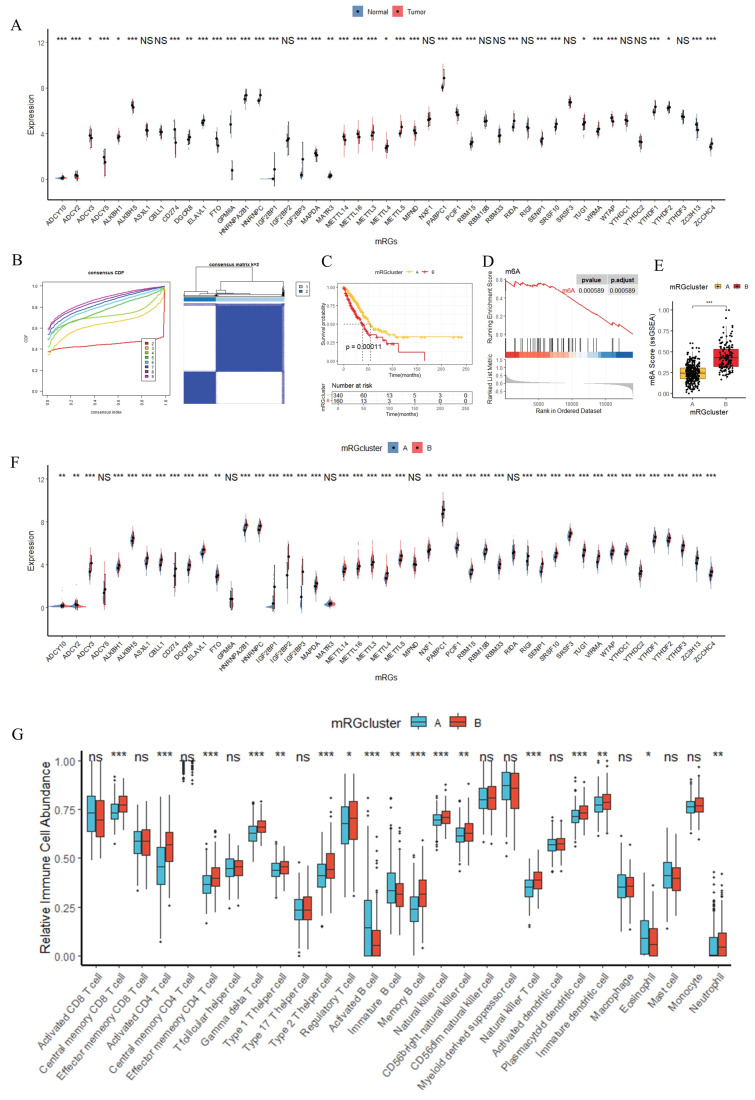
** Identification of mRGclusters and Analysis of Their Molecular Characteristics, Clinical Prognosis, and Immune Infiltration Landscape.** (A) and (F) Differential expression analysis of mRGs between Normal and Tumor groups (A) and between mRGcluster A and B (F). (B) Identification of two distinct clusters via unsupervised clustering analysis of 47 mRGs. (C) KM analysis of OS probability in LUAD patients stratified by mRGclusters. (D) GSEA analysis of the m6A signaling pathway. (E) Comparison of m6A scores between mRGCluster A and B. (G) Immune cell infiltration landscape in mRGcluster A (blue) vs B (red) quantified via ssGSEA. *P < 0.05; **P < 0.01; ***P < 0.001; ns, not significant.

Figure 1 illustrates the study workflow. To characterize the expression patterns of MRGs and their associations with prognosis, m6A pathways, and immune infiltration in LUAD, comprehensive analyses were conducted as shown in Figure 2. Figure 2A demonstrated that mRGs exhibited significantly differential expression between normal and tumor samples in the TCGA-LUAD dataset, highlighting the dysregulation of m6A regulatory machinery during LUAD carcinogenesis. Using the expression matrix of mRGs, consensus clustering in Figure 2B (Supplementary Figure S1) stratified all TCGA-LUAD samples into two distinct mRGclusters (A and B). Survival analysis in Figure 2C revealed that patients in mRGcluster B had significantly worse overall survival than those in cluster A (*P* < 0.05), indicating that mRG expression patterns drive prognostic heterogeneity in LUAD. To explore alterations in m6A pathways, Figure 2D shows GSEA analysis using the 47 MRGs as a custom gene set to evaluate m6A-related pathway activation differences between mRGcluster B and mRGcluster A. Consistent with this, ssGSEA in Figure 2E further confirmed that m6A pathway scores were significantly higher in mRGcluster B compared to cluster A, reinforcing the differential m6A pathway activity between the two clusters. Figure 2F visualized the expression of individual mRGs between clusters A and B, where distinct expression patterns of mRGs were observed—an outcome that aligns with the clustering basis and pathway activation patterns. Finally, immune infiltration analysis in Figure 2G revealed significant differences in the relative abundance of multiple immune cell subsets (e.g., activated CD4+ T cells, activated B cells, natural killer T cells) between mRGcluster A and mRGcluster B. Collectively, these results underscore the critical role of mRG-based clustering in distinguishing LUAD subgroups with differential prognosis, m6A pathway activity, and immune microenvironment features.

### Multi-layered Functional Enrichment Analyses of mRGclusters and LUAD-Relevant Signatures

**Figure 3:**
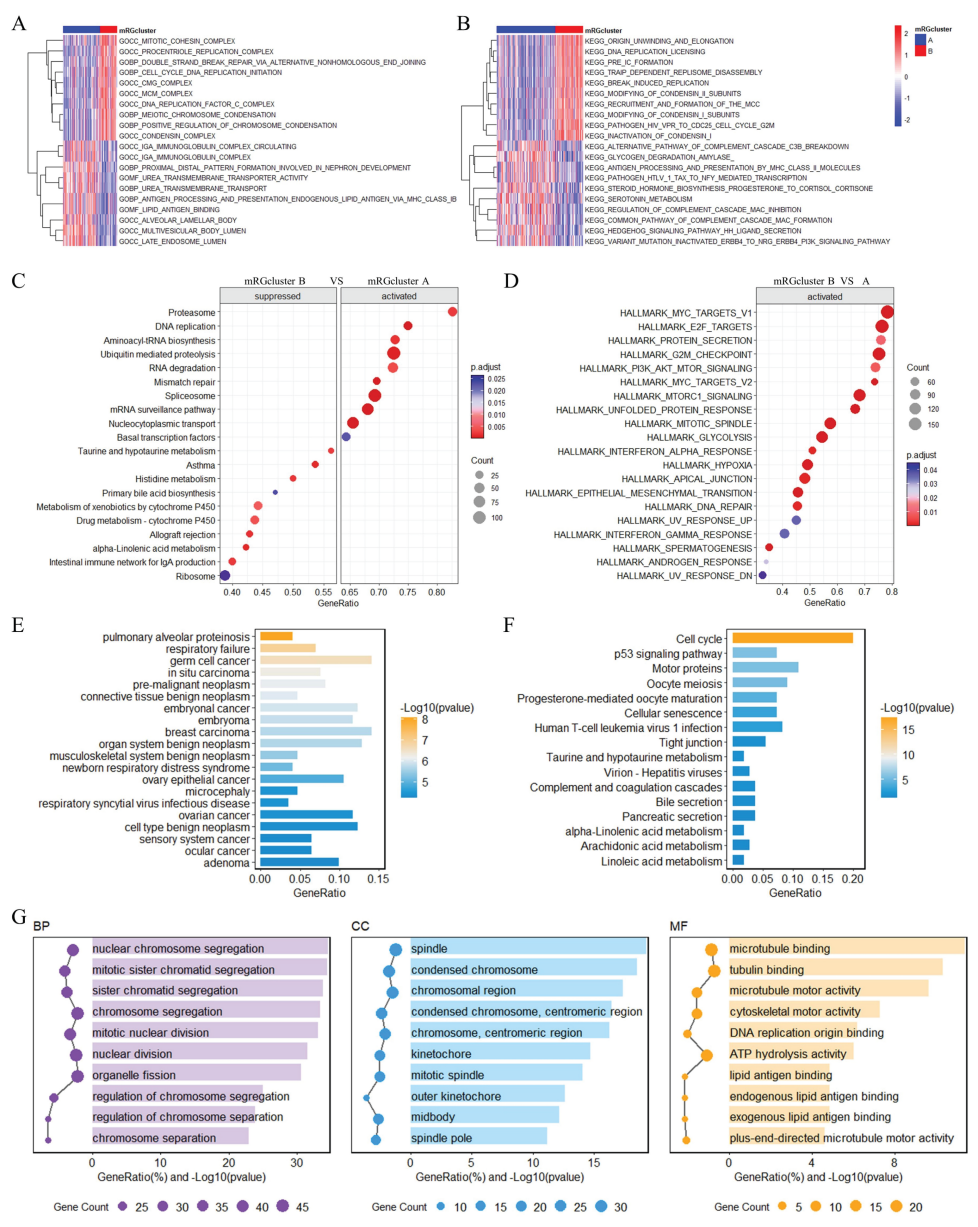
** Multi-layered Functional Enrichment Analyses of mRGclusters in LUAD.** GO (A) and KEGG (B) GSVA enrichment analyses between mRGcluster A and B. GSEA analysis of KEGG pathways (C) and hallmark terms (D) between mRGcluster A and B. DO (E), KEGG (F), and GO (G) enrichment analyses highlighting DEG variations between the two mRGclusters.

To delineate functional disparities associated with mRGclusters, we performed multi-layered functional enrichment analyses (Figure 3). GSVA analysis revealed that, in mRGcluster B versus A, the top 10 enriched Gene Ontology (GO) terms (Figure 3A) prominently encompassed processes related to immune response regulation and metabolic process modulation, whereas the top 10 Kyoto Encyclopedia of Genes and Genomes (KEGG) pathways (Figure 3B) were significantly enriched in cell cycle progression and the PI3K-Akt signaling cascade. GSEA further demonstrated that KEGG pathways including cell cycle and DNA replication were highly enriched in mRGcluster B, while processes like proteasome and aminoacyl-tRNA biosynthesis were suppressed in mRGcluster B (Figure 3C). For Hallmark gene sets (Figure 3D), mRGcluster B exhibited activation of MYC_TARGETS_V1/V2, E2F_TARGETS, OXIDATIVE_PHOSPHORYLATION, and UNFOLDED_PROTEIN_RESPONSE—reflecting enhanced cell proliferation, metabolic reprogramming, and stress response compared to mRGcluster A. Moreover, Disease Ontology (DO) analysis of differential genes between mRGcluster B and A (Figure 3E) highlighted enrichments in pulmonary diseases (e.g., pulmonary alveolar proteinosis, respiratory failure) and epithelial-derived cancers (e.g., pre-malignant neoplasm, breast carcinoma, embryonal cancer), which aligns with the pathology of LUAD. KEGG analysis of these differential genes (Figure 3F) showed significant enrichment in pathways such as cell cycle, p53 signaling pathway, oocyte meiosis, and metabolic cascades like taurine and hypotaurine metabolism and complement and coagulation cascades. And GO analysis (Figure 3G) dissected functional differences at three levels: in biological processes (BP), terms like nuclear chromosome segregation, mitotic sister chromatid segregation, and nuclear division dominated, emphasizing mitotic cell cycle progression; in cellular components (CC), spindle, condensed chromosome, kinetochore, and centromeric regions were enriched, reflecting structural elements supporting cell division; for molecular functions (MF), microtubule binding, ATP hydrolysis activity, and cytoskeletal motor activity were prominent, underscoring molecular interactions driving cellular dynamics.

Collectively, these findings elucidate that mRGcluster B is characterized by intensified cell cycle progression, DNA replication, and metabolic rewiring, while also being linked to LUAD-relevant pathological and immunological signatures—providing a functional framework for mRGcluster-mediated heterogeneity in LUAD.

### Construction and Validation of a Prognostic Model for LUAD

**Figure 4:**
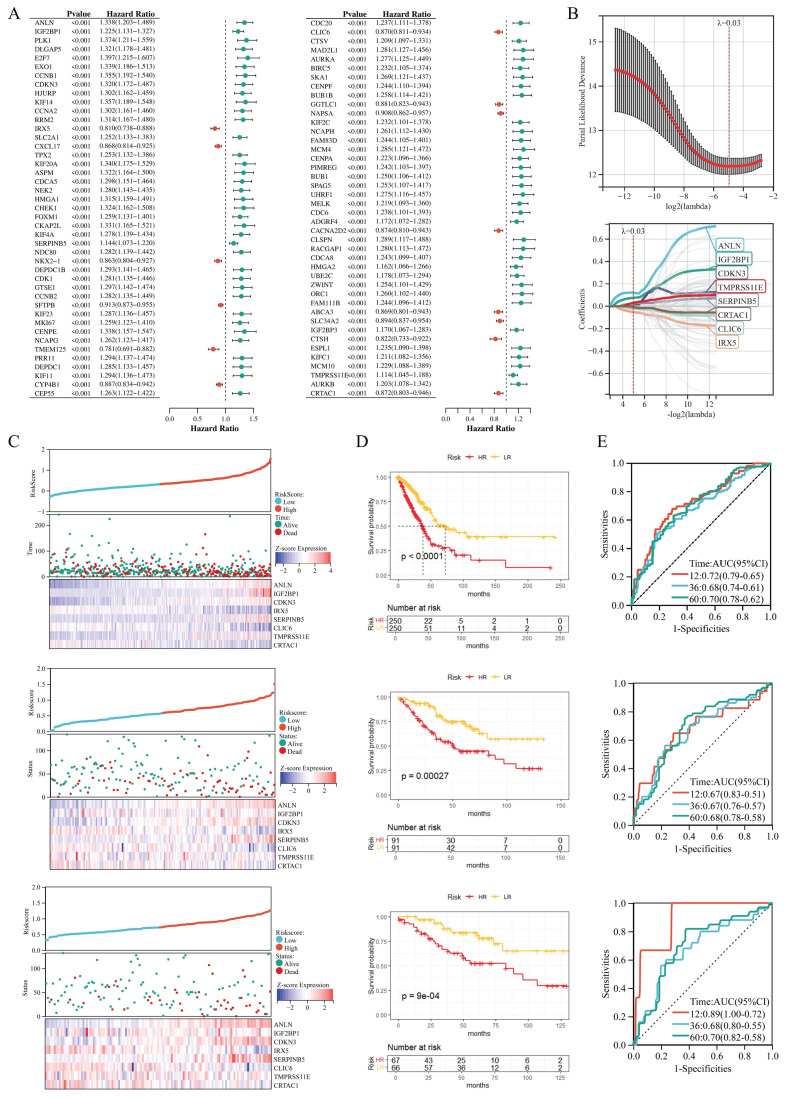
** Construction and Validation of a Prognostic Model for LUAD.** (A) Univariate Cox regression analysis of DEGs between mRGcluster A and B, presenting hazard ratios and P-values for genes associated with OS. (B) LASSO regression analysis of 85 prognosis-associated genes to establish the m6A related risk score. (C) Distributions of risk scores, patient survival status, and expression heatmaps of the 8 signature genes across three datasets (TCGA-LUAD, GSE41271, GSE42127). Each subplot includes risk scores (top), survival status (middle), and gene expression patterns (bottom). (D) KM survival curves comparing OS between HR and LR groups in the three datasets. (E) Time-dependent ROC curves evaluating the model's predictive accuracy for 1-, 3-, and 5-year OS in the three datasets, with corresponding AUC values and 95% confidence intervals.

In constructing a prognostic model for LUAD, we first performed univariate Cox regression analysis on 215 DEGs (Supplementary Table S2), which identified 85 prognostic genes, with Figure 4A displaying the hazard ratios for these genes. Subsequently, LASSO Cox regression analysis was conducted on these 85 prognostic genes with 10-fold cross-validation to obtain the optimal model; as shown in Figure 4B, setting the lambda value at 0.0320774136658451 yielded a model comprising 8 signature genes, with the formula: RiskScore = 0.120644340741998×ANLN + 0.0756323386868951×IGF2BP1 + 0.0029139181750111×CDKN3 - 0.0514445484796121×IRX5 + 0.0255764686431857×SERPINB5 - 0.0207518111783162×CLIC6 + 0.0297086707424968×TMPRSS11E - 0.00682769031303758×CRTAC1. Risk scores for all patients are provided in Supplementary Table S3. Patients were stratified into HR and LR groups based on the 50th percentile of RiskScore. Furthermore, Figure 4C-E illustrate the risk plots, KM survival curves, and ROC curves across three datasets (TCGA-LUAD, GSE41271, GSE42127): the KM curves demonstrated that the HR group had significantly worse overall survival compared to the LR group (all *P* < 0.05), and the ROC curves exhibited favorable areas under the curve (AUC) values (e.g., the AUC at 1 year ranged from ~0.7 to ~0.9 across datasets), indicating robust prognostic predictive ability; meanwhile, we analyzed the relationships between risk scores, patients' follow-up time, events, and expression changes of each of the 8 signature genes: as risk scores increased (x-axis from left to right in the top risk plots), patients' survival rates decreased significantly (middle KM panels); consistently, *IRX5*, *CLIC6*, and *CRTAC1* (protective factors) showed downregulated expression with increasing risk scores, while *ANLN*, *IGF2BP1*, *CDKN3*, *SERPINB5*, and *TMPRSS11E* (risk factors) exhibited upregulated expression with higher risk scores.

### Functional and Immune Analyses of HR/LR Groups and Their Association with the Prognostic Signature and RiskScore in LUAD

**Figure 5:**
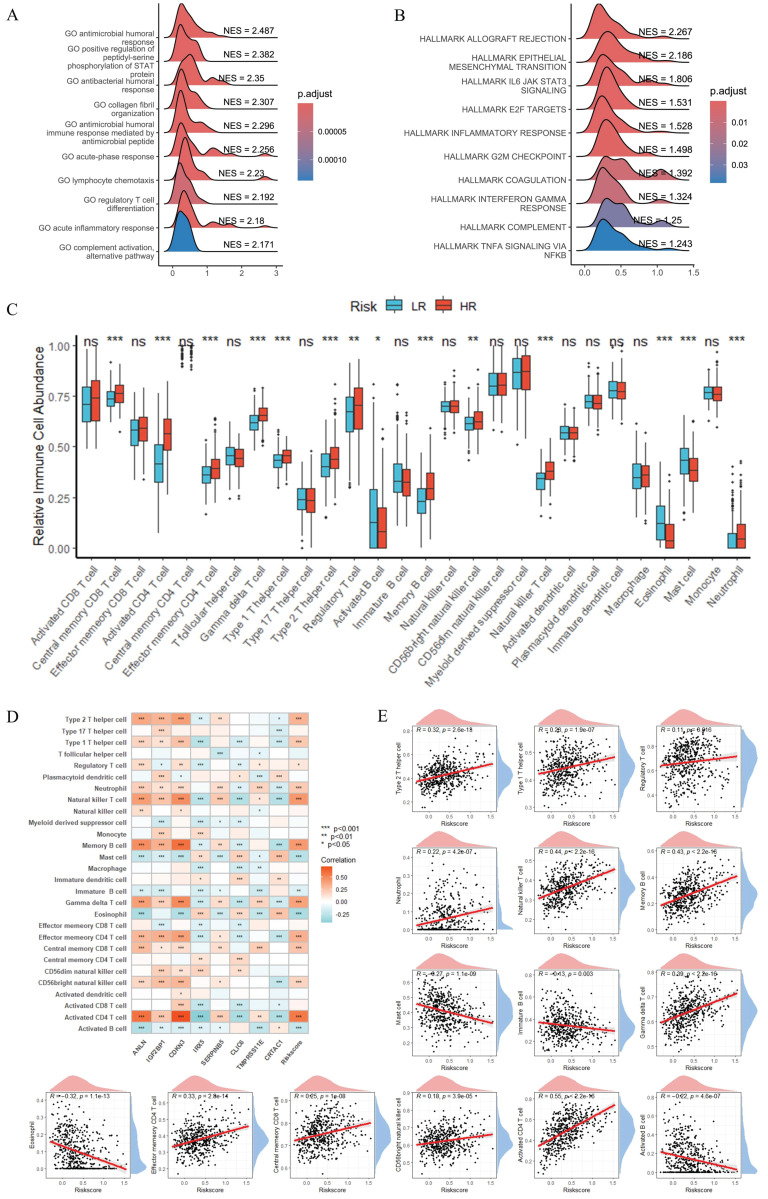
** Functional and Immune Analyses of HR/LR Groups and Their Association with the Prognostic Signature and RiskScore in LUAD.** GSEA of GO terms (A) and Hallmark gene sets (B), showing functional enrichment differences between HR and LR groups. (C) Boxplots of relative immune cell abundances, comparing LR and HR groups. (D) Correlation heatmap between the 8 signature genes, RiskScore, and 28 immune cell types, with color gradients representing Pearson correlation coefficients. (E) Scatter plots showing the correlation between RiskScore and immune cells. *P < 0.05; **P < 0.01; ***P < 0.001; ns, not significant.

To explore the functional and immune features of HR and LR groups and their links to the prognostic signature and RiskScore, we performed subsequent analyses (Figure 5). GSEA revealed that GO terms related to antimicrobial humoral response and inflammatory response were enriched in the HR group compared to the LR group (Figure 5A), while Hallmark gene set analysis (Figure 5B) demonstrated significant activation of signatures including immune checkpoint, epithelial-mesenchymal transition (EMT), and IL-6/JAK/STAT3 signaling in the HR group. Immune infiltration analysis (Figure 5C) showed notable differences in the relative abundance of multiple immune cell subsets between HR and LR groups (e.g., activated CD4⁺ T cells and natural killer cells exhibited distinct distributions). Furthermore, correlation analysis between the 8 signature genes, RiskScore, and 28 immune cell types (Figure 5D) uncovered diverse correlation patterns; Figure 5E specifically illustrated the correlations between RiskScore and immune cells, showing that RiskScore had a strong positive correlation with activated CD4⁺ T cells, natural killer (NK) cells and memory B cells (r > 0.4, *P* < 0.001) and a strong negative correlation with mast cells, eosinophils and activated B cells (r < -0.2, *P* < 0.001). Collectively, these results highlight the interplay between the prognostic gene signature, RiskScore, and the tumor immune microenvironment, with RiskScore closely linked to the polarization of pro- and anti-tumor immune subsets in LUAD.

### Characterization of Signature Genes: Expression Patterns, Clinical Correlations, and Prognostic Implications in LUAD

**Figure 6:**
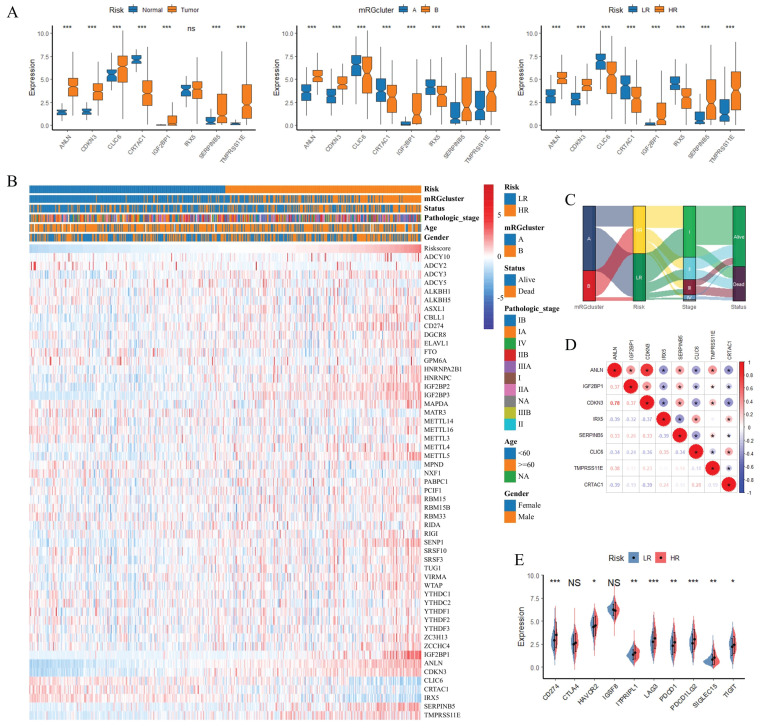
** Characterization of Signature Genes: Expression Patterns, Clinical Correlations, and Prognostic Implications in LUAD.** (A) Boxplots showing the expression of 8 signature genes across three comparisons: normal vs. tumor, mRGcluster A vs. B, and LR vs. HR groups in TCGA-LUAD. (B) Heatmap integrating clinical information (risk status, mRGcluster, pathologic stage, age, gender, survival status), expression of 47 MRGs, and 8 signature genes, illustrating distinct expression profiles across subgroups and clinical parameters. (C) Sankey diagram visualizing associations among mRGcluster, risk group, pathologic stage, and survival status. (D) Correlation heatmap of pairwise relationships among the 8 signature genes. (E) Boxplots of immune checkpoint gene expression in LR vs. HR groups. *P < 0.05; **P < 0.01; ***P < 0.001; ns, not significant.

For further characterization of the signature genes and their associations with clinical features, mRGs, and immune contexture in LUAD, we conducted comprehensive analyses (Figure 6). Figure 6A displayed boxplots of the eight signature genes' expression, revealing significant differences across normal versus tumor samples, mRGcluster A versus B, and LR versus HR groups in TCGA-LUAD—with most signature genes showing altered expression patterns that distinguished tumor tissues, mRGcluster B, and HR groups, indicating their potential involvement in LUAD progression and risk stratification. Figure 6B presented a heatmap integrating clinical information (including risk status, mRGcluster, pathologic stage, age, gender, and survival status), the expression of 47 mRGs, and the eight signature genes, where distinct expression profiles of mRGs and signature genes were observed between different risk groups and mRGclusters, and these profiles also correlated with clinical parameters such as pathologic stage. Moreover, the Sankey diagram in Figure 6C visualized the associations among mRGcluster, risk group, pathologic stage, and survival status, illustrating how mRG-based clustering and risk stratification intertwined with clinical outcomes.

The correlation heatmap in Figure 6D quantified pairwise correlations among the eight signature genes, showing notable positive or negative correlations (e.g., strong positive correlations among *ANLN*, *IGF2BP1*, and *CDKN3*) that reflected their coordinated regulatory roles. Figure 6E's boxplots depicted the expression of immune checkpoint genes in LR versus HR groups, with several checkpoints (e.g., *LAG3*, *PDCD1*) exhibiting higher expression in HR groups, suggesting an immunosuppressive microenvironment in HR patients. Additionally, KM survival curves in Supplementary Figure S2 verified the prognostic value of each individual signature gene: high expression of *CRTAC1*, *CLIC6*, and *IRX5* was associated with favorable overall survival, whereas high expression of *ANLN*, *IGF2BP1*, *CDKN3*, *SERPINB5*, and *TMPRSS11E* correlated with worse overall survival (*P* < 0.05), which was consistent with the survival pattern observed in Figure 4C. Collectively, these results underscored that the eight signature genes not only distinguished tumor from normal tissues and different molecular/clinical subgroups but also correlated with immune checkpoint expression and predicted patient survival, highlighting their multifaceted roles in LUAD biology and clinical outcomes.

### TMB and *CSMD3* Mutation: Impact on Risk Stratification, Immune Infiltration, and Survival in LUAD

**Figure 7:**
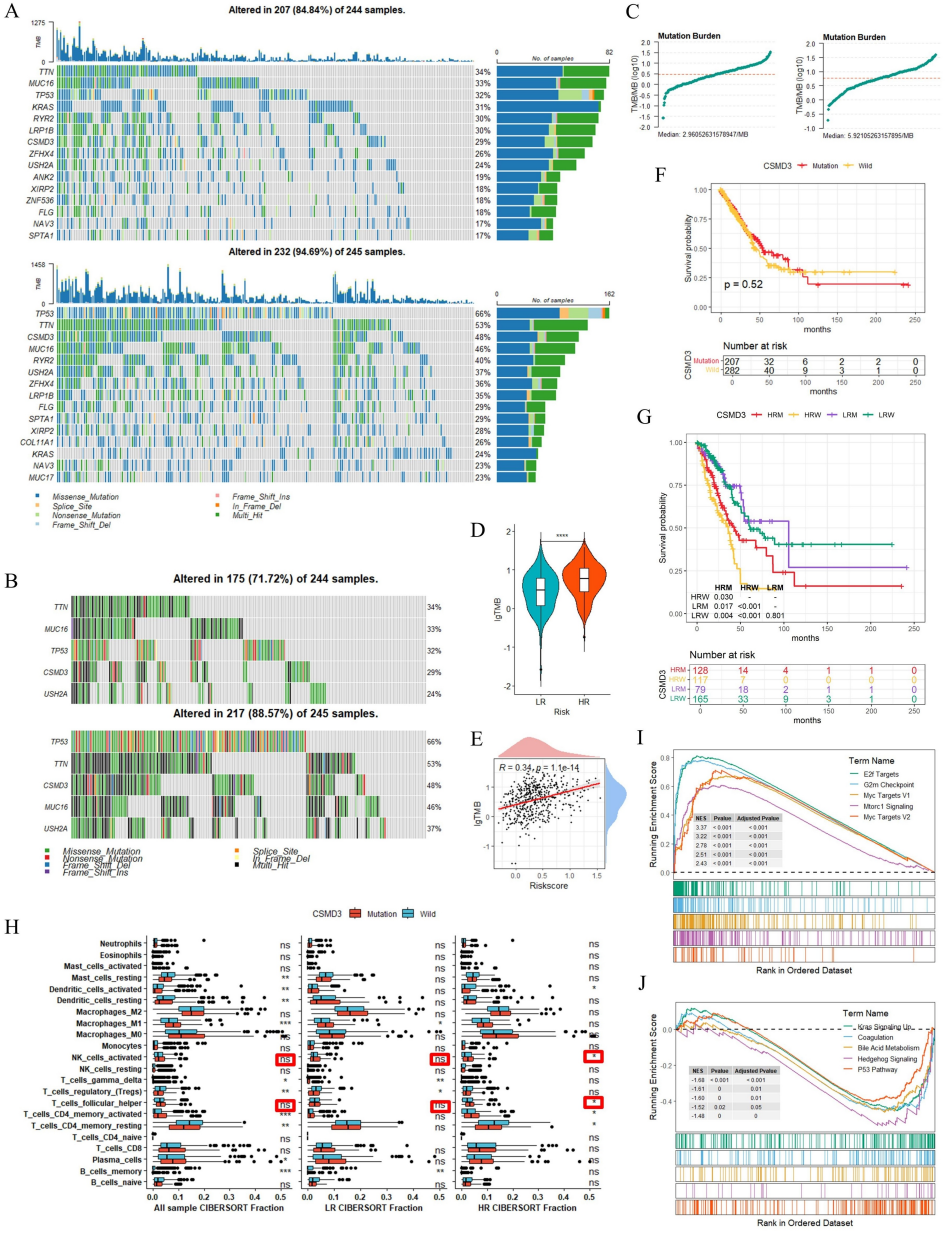
** TMB and *CSMD3* Mutation: Impact on Risk Stratification, Immune Infiltration, and Survival in LUAD.** TMB waterfall plots, depicting genetic alterations (mutation types, frequencies) in LR/HR groups (A) and mRGclusters (B). (C) Trends of mutation burden in LR and HR groups. (D) Boxplot of TMB comparing LR and HR groups. (E) Scatter plot illustrating the correlation between TMB and RiskScore. (F) KM survival curve comparing OS between *CSMD3*-mutated and wild-type groups. (G) KM survival curves and sample distribution for four subgroups (LR with *CSMD3* mutation (LRM), LR wild-type (LRW), HR mutated (HRM), HR wild-type (HRW)). (H) Boxplots of immune cell abundances (via CIBERSORT) comparing *CSMD3*-mutated and wild-type groups across all samples, LR, and HR subgroups. (I) GSEA Hallmark plots, showing pathway dysregulation associated with *CSMD3* mutation. *P < 0.05; **P < 0.01; ***P < 0.001; ns, not significant.

To elucidate the TMB characteristics and molecular alterations associated with risk stratification, we performed the relevant analyses (Figure 7). Figure 7A presented TMB waterfall plots for the LR and HR groups: among 244 samples, 207 (84.8%) in LR group and 232 (94.9%) in the HR group harbored genetic alterations. The top mutated genes (e.g., *TP53*, *TTN*, *MUC16*) exhibited diverse mutation types and distinct frequency distributions, and the stacked bar plots further quantified the proportional mutation distribution of each gene across subgroups. Focusing on the top 5 genes with divergent mutation rates between the two risk groups, Figure 7B showed that *CSMD3* and several other genes displayed higher mutation frequencies in the HR cohort. Figure 7C summarized the TMB distribution trends, and Figure 7D indicated that the HR group had significantly higher log-transformed TMB (lgTMB) than the LR group (*P* < 0.001). Figure 7E's scatter plot showed a weak positive correlation between lgTMB and RiskScore (r = 0.34, *P* < 0.001), suggesting an association between elevated RiskScore and increased TMB. Survival analyses of the top 5 mutated genes versus wild-type status are provided in Supplementary Figure S3. For *CSMD3*, no significant survival difference was detected between mutated and wild-type populations in the overall cohort (*P* = 0.52; Figure 7F). However, subgroup stratification (LR with *CSMD3* mutation (LRM), LR wild-type (LRW), HR mutated (HRM), HR wild-type (HRW)) revealed that HRM patients exhibited better overall survival than HRW patients (*P* < 0.05), whereas no noticeable survival difference was observed with the LR subgroups (Figure 7G). Immune infiltration analysis using CIBERSORT revealed a marginal increase in the abundances of activated NK cells and follicular helper T cells in HRM patients relative to HRW patients, while no obvious immunological discrepancy was detected between LRM and LRW subgroups (Figure 7H). Such transcriptome-based immune alterations were observed merely within the high-risk subgroup. GSEA was further performed to explore altered biological pathways between *CSMD3*-mutated and wild-type samples (Figures 7I and 7J). The upregulated pathways included MTORC1 SIGNALING and OXIDATIVE PHOSPHORYLATION, whereas downregulated pathways contained APICAL JUNCTION and INTERFERON GAMMA RESPONSE. Collectively, these observational results preliminarily indicated that TMB and *CSMD3* mutation status may correlate with risk stratification, immune cell composition, and pathway alterations in LUAD. The survival benefit of CSMD3 mutation was specifically limited to the high-risk subgroup, which represents an exploratory subgroup-specific observation.

### RiskScore-Based Prognostic Nomogram for LUAD: Calibration and Discrimination Across Cohorts

**Figure 8:**
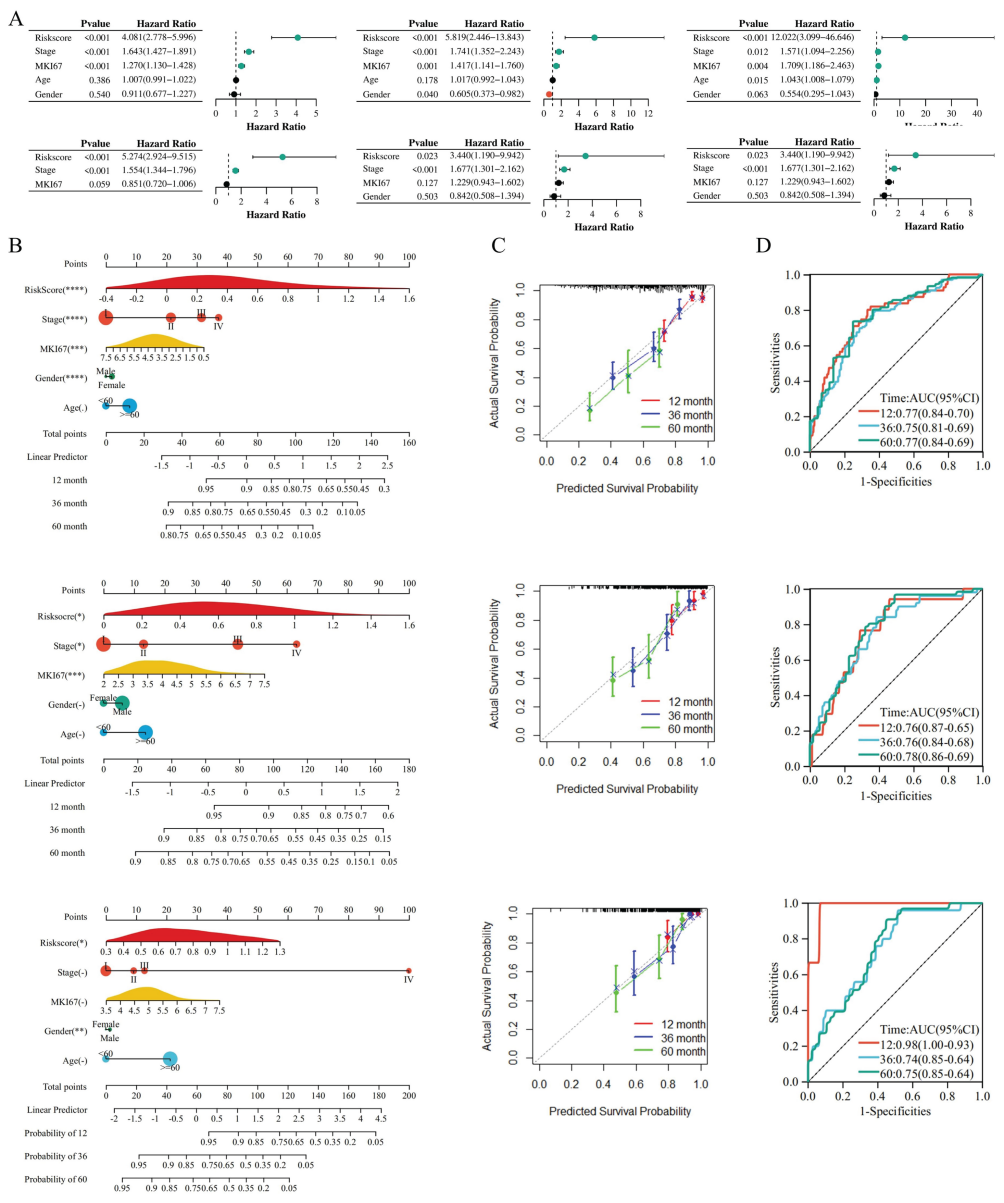
** RiskScore-Based Prognostic Nomogram for LUAD: Calibration and Discrimination Across Cohorts.** (A) Univariate and multivariate Cox regression analyses across TCGA-LUAD, GSE41271, and GSE42127 datasets, displaying hazard ratios and P-values for RiskScore and clinical features (stage, age, gender, *MKI67*). (B) Nomograms integrating RiskScore, clinical stage, *MKI67*, age, and gender to predict 12-, 36-, and 60-month survival probabilities for the three datasets. (C) Calibration curves comparing predicted and actual survival probabilities at 12, 36, and 60 months across datasets. (D) Time-dependent ROC curves evaluating the model's predictive accuracy for 12-, 36-, and 60-month overall survival. *P < 0.05; **P < 0.01; ***P < 0.001; ns, not significant.

When evaluating the prognostic value of RiskScore and constructing a predictive model, we performed Cox regression analyses and nomogram-based assessments across multiple datasets (Figure 8). Figure 8A presented univariate and multivariate Cox regression results for TCGA-LUAD, GSE41271, and GSE42127, where RiskScore consistently exhibited a significant hazard ratio (HR) with *P* < 0.001 in univariate analysis and remained an independent prognostic factor in multivariate models (after adjusting for stage, *MKI67*, age, and gender), confirming its robust prognostic significance. The RiskScore provided independent and incremental prognostic value beyond conventional clinical features and the proliferation marker *MKI67*. *MKI67* was included in the primary nomogram to enhance prognostic discrimination, whereas we acknowledge it is not a uniformly standardized indicator in routine LUAD clinical practice.

We further constructed a simplified nomogram excluding *MKI67* that integrates only routinely accessible clinical variables (age, gender, tumor stage) and RiskScore, verifying that RiskScore still maintained independent prognostic significance and stable predictive performance. Subsequently, Figure 8B displayed nomograms integrating RiskScore, clinical stage, *MKI67*, age, and gender for the three datasets, with points assigned to each variable (RiskScore contributing the most weight, as indicated by the longest axis) to predict 12-, 36-, and 60-month survival probabilities, thus facilitating individualized risk stratification. To assess model calibration, Figure 8C's calibration curves compared predicted survival probabilities with actual outcomes at 12, 36, and 60 months across datasets, showing close alignment (points clustered near the 45° diagonal line), which indicated high consistency between prediction and reality. Finally, Figure 8D's ROC curves quantified the model's predictive accuracy, with AUC values for 12-month survival ranging from 0.70 to 0.77, 36-month from 0.70 to 0.81, and 60-month from 0.70 to 0.84 across datasets, demonstrating favorable discriminative ability for long-term survival. Collectively, these results validated that the RiskScore-based nomogram could reliably predict prognosis in LUAD, with strong calibration and discrimination across independent cohorts.

### RiskScore as a Predictor of Immunotherapy Response and Chemotherapy Sensitivity in LUAD

**Figure 9:**
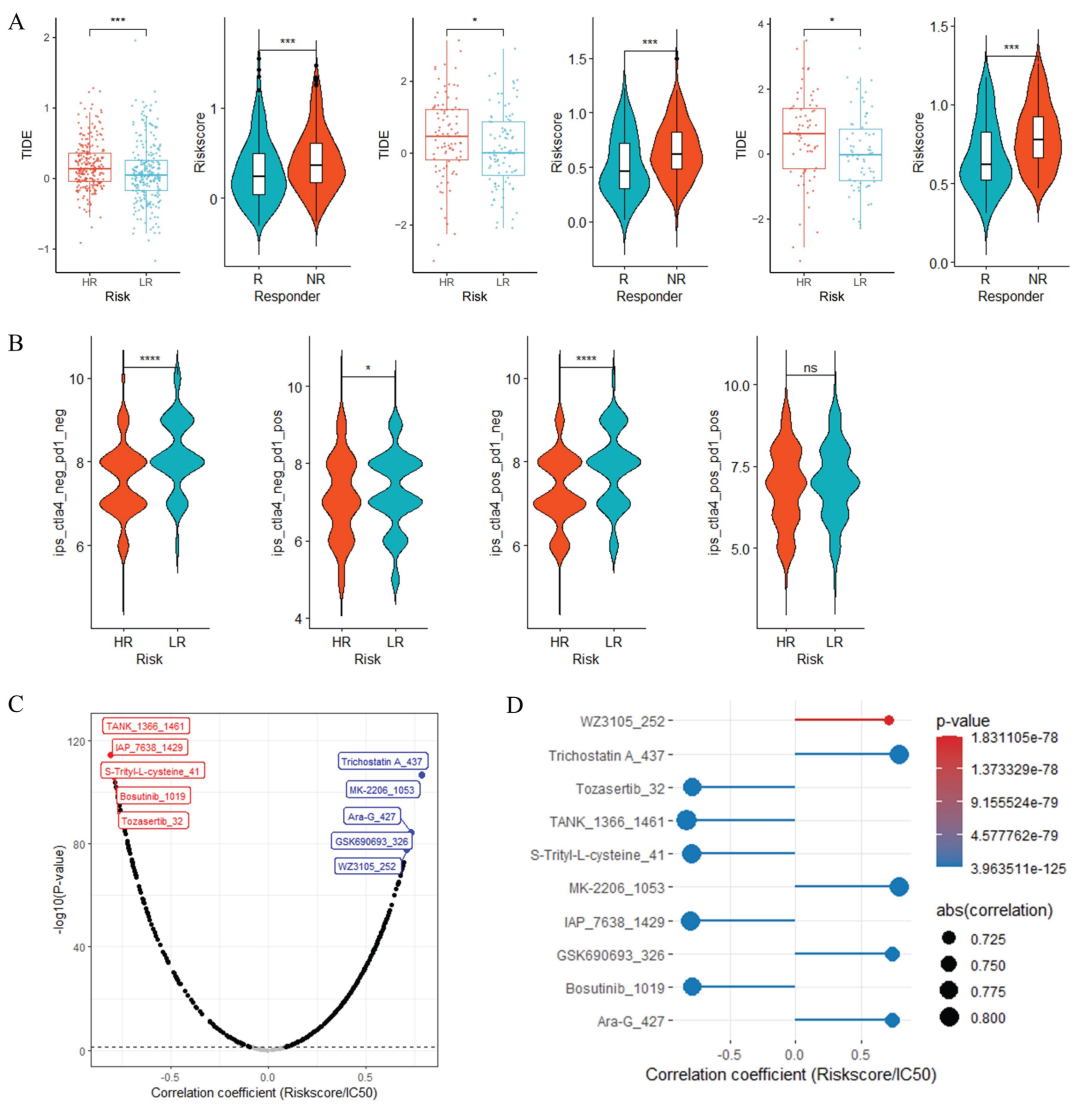
** RiskScore as a Predictor of Immunotherapy Response and Chemotherapy Sensitivity in LUAD.** (A) Comparisons of TIDE scores between HR and LR groups, and RiskScore between immunotherapy responders (R) and non-responders (NR) across three datasets. (B) Boxplots of IPS in HR vs. LR groups from the TCGA-LUAD dataset. Correlation trend plots (C) and detailed results (D) of chemotherapy drug IC50 with RiskScore, highlighting top 5 drugs with negative (red) and positive (blue) correlations. *P < 0.05; **P < 0.01; ***P < 0.001; ns, not significant.

When investigating the potential associations of RiskScore with immunotherapy response and chemotherapy sensitivity, we performed bioinformatic prediction analyses based on TIDE, IPS and oncoPredict algorithms (Figure 9). In Figure 9A, across three datasets (TCGA-LUAD, GSE41271, GSE42127), TIDE scores (Supplementary Table S4) were significantly higher in HR groups than in LR groups (***, *P* < 0.001); meanwhile, predicted immunotherapy responders (R) had a significantly lower RiskScore compared to predicted non-responders (NR), which collectively suggested that HR group tended to display enhanced immune evasion characteristics and may have a poorer potential immunotherapy response tendency, while patients with lower RiskScore might be more inclined to derive theoretical immunotherapy benefit. Figure 9B demonstrated that in the TCGA-LUAD dataset, Immune Phenotype Scores (IPS, Supplementary Table S5), including subscores such as ips_ctla4 and ips_pd1, were markedly lower in HR groups than in LR groups, indicating that the LR group possessed a more favorable immune phenotype that may confer theoretical advantages for immunotherapy efficacy prediction. For chemotherapy sensitivity analysis, Figure 9C presented correlation trend plots of chemotherapy drug IC50 (Supplementary Table S6) with RiskScore, where red and blue boxes respectively illustrated the top 5 drugs with negative and positive correlations with RiskScore; Figure 9D further displayed the correlation results of these top 5 drugs with RiskScore. These analyses revealed two distinct correlational patterns: the top 5 drugs negatively correlated with RiskScore (TANK_1366_1461, IAP_7638_1429, S-Trityl-L-cysteine_41, Bosutinib_1019 and Tozasertib_32) showed a decreasing trend in predicted IC50 as RiskScore increased, implying that HR group may theoretically be more sensitive to these agents; in contrast, the top 5 drugs positively correlated with RiskScore (Trichostatin-A_437, MK-2206_1053, Ara-G_427, GSK690693_326 and WZ3105_252) exhibited a decrease in predicted IC50 as RiskScore decreased, indicating that the LR group may have theoretical sensitivity advantages to these positively correlated chemotherapy drugs. Supplementary Figure S4A's violin plots further confirmed extremely significant intergroup differences in predicted IC50 of these top 5 drugs between LR and HR groups (*P* < 0.001), consistent with their correlation trends with RiskScore. Moreover, Supplementary Figure S4B's scatter plots verified that the predicted IC50 of these top 5 drugs was closely correlated with RiskScore, showing strong correlation strength (|r|> 0.7, *P* < 0.001). Collectively, these bioinformatic predictions indicated that RiskScore could serve as a potential biomarker to infer differential therapeutic vulnerability to immunotherapy and chemotherapy in LUAD, providing hypothesis-generating clues for subsequent experimental and clinical verification.

### ScRNA Sequencing Uncovers TME Cellular Composition, Signature Gene Expression, and Altered Cell-Cell Crosstalk in LUAD

**Figure 10:**
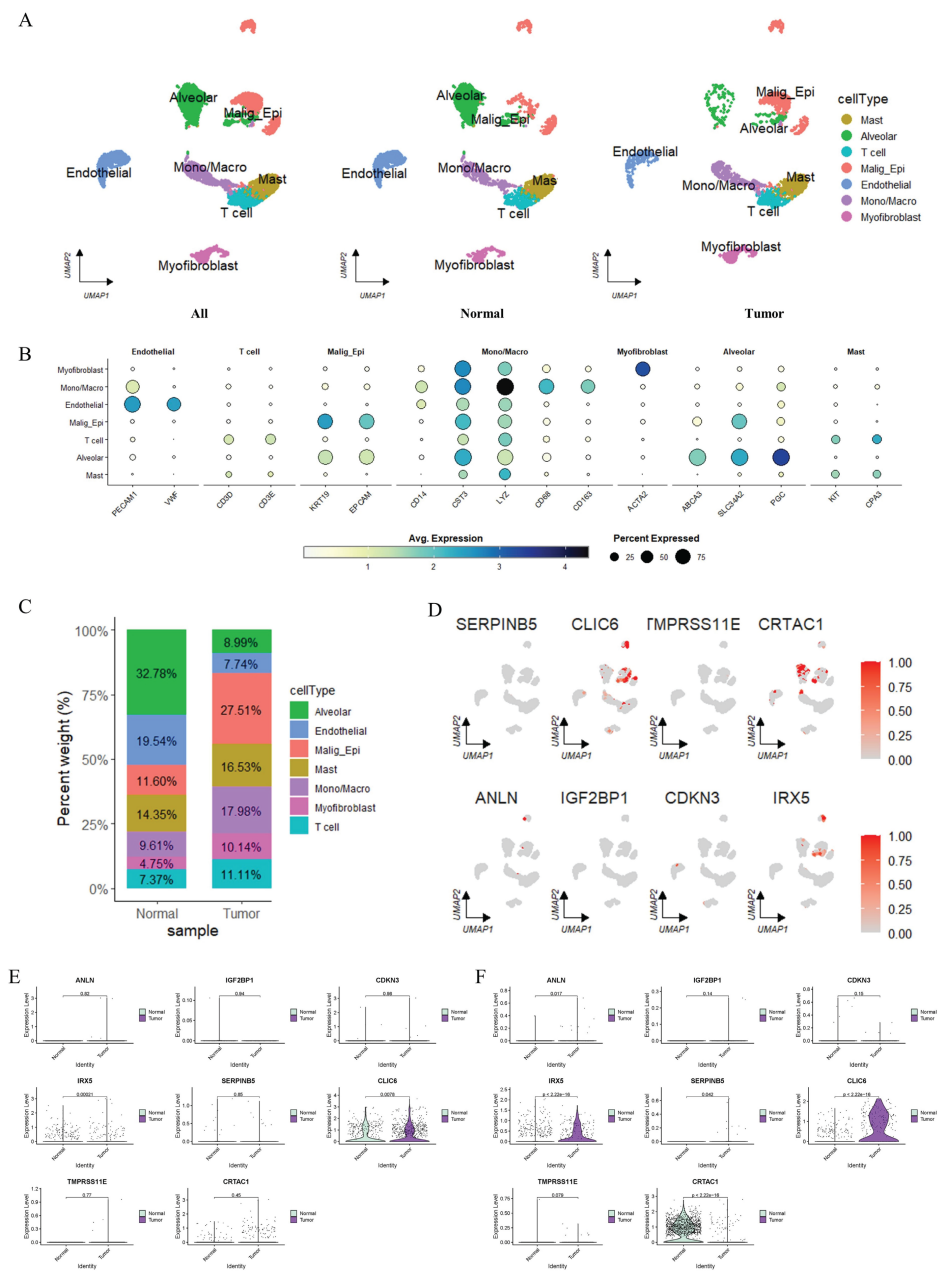
** Single-cell RNA Sequencing Analysis.** (A) UMAP visualization of single-cell annotations stratified by adjacent normal lung tissue and primary tumor tissue. Cell labels were defined on the integrated dataset and kept consistent across tissue subgroups. Malignant epithelial cells (Malign_Epi) were predominantly enriched in tumor tissue. The sporadic Malign_Epi cells observed in adjacent normal tissue likely represent pre-neoplastic field-effect alterations rather than genuine tumor cell contamination. (B) Heatmap of marker gene expression across cell types. (C) Stacked bar plot quantifying the percentage of each cell type in normal and tumor samples. (D) Differential expression analysis of 8 signature genes in distinct cell clusters. (E-F) Violin plots comparing the expression of eight signature genes in Malig_Epi cells (E) and Alveolar epithelial cells (F) between normal adjacent and tumor tissue samples. Sporadic Malign_Epi cells detected in normal tissue were excluded from the comparison to ensure pure and reliable expression profiling of the two cell populations.

To dissect the cellular composition and functional heterogeneity in the TME, we performed scRNA sequencing analyses (Figure 10). Uniform Manifold Approximation and Projection (UMAP) visualization in Supplementary Figure S5A revealed Seurat-defined clusters, while Figure 10A annotated cell types (Alveolar, T cell, Malig_Epi, Endothelial, Mono/Macro, Myofibroblast, Mast, etc.) in normal and tumor samples, showing distinct spatial distribution patterns between these two groups. The heatmap in Figure 10B illustrated the expression levels of marker genes across different cell types, where color gradients represented average gene expression and dot size indicated the proportion of cells expressing each marker, thus validating cell type identities (e.g., T cell markers showed high expression in T cells, and alveolar-specific markers were enriched in Alveolar cells). The stacked bar plot in Figure 10C quantified cell type proportions, demonstrating that Malig_Epi cells were more abundant in tumors whereas Alveolar cells were enriched in normal samples. For differential gene analysis, Supplementary Figure S5B's volcano plot displayed significantly upregulated (orange) and downregulated (purple) genes across clusters, reflecting cluster-specific transcriptional programs. Focusing on signature genes, Figure 10D's UMAP plots visualized the expression of eight key genes (*ANLN*, *IGF2BP1*, *CDKN3*, *IRX5*, *SERPINB5*, *CLIC6*, *TMPRSS11E*, *CRTAC1*) across cell clusters, with prominent expression in Malig_Epi and other lineages. Moreover, Figures 10E and 10F compared the expression of these 8 signature genes in Malig_Epi and Alveolar cells, respectively, between normal and tumor groups. In Alveolar cells (Figure 10F), genes like CLIC6 were markedly upregulated in tumor-derived cells, whereas CRTAC1 was markedly downregulated. Additionally, the expression patterns of signature genes in Malig_Epi cells (Figure 10E) also differed between normal and tumor states, highlighting tumor-specific transcriptional alterations in both malignant and non-malignant cell populations. Finally, Supplementary Figure S5C's cell-cell interaction networks showed that both the number of interactions and interaction weights differed between normal and tumor tissues, with remodeled communication patterns (e.g., altered interactions involving T cells, Alveolar cells, and Malig_Epi cells) in the TME. Collectively, these results elucidated the cellular hierarchy, signature gene expression specificity, and disrupted cell-cell crosstalk in LUAD at single-cell resolution.

## Discussion

This study highlights that m6A methylation, modulated by methyltransferases, demethylases and m6A-binding proteins, serves as a core epigenetic regulator of LUAD progression 14,15. We identified two distinct mRGclusters (A/B) with divergent prognosis, m6A pathway activity, and immune infiltration. The dynamic and reversible nature of m6A modification enables post-transcriptional tuning of gene expression: mRGcluster B presented elevated m6A pathway activity, which probably arises from a disrupted balance between m6A writers and erasers. Such imbalance may upregulate pro-tumorigenic genes and repress tumor suppressors, consistent with previous LUAD reports 15,16. Aberrant m6A alteration is not only a hallmark of tumor heterogeneity but also a driver of clinical outcomes. Patients in mRGcluster B exhibited inferior overall survival, indicating that dysregulated m6A modification correlates with more aggressive LUAD phenotypes. This prognostic discrepancy is partially attributed to m6A-mediated remodeling of the TME, with distinct infiltration patterns of activated CD4⁺ T cells and NKT cells observed between subtypes. Targeting m6A-related pathways may remodel the immunosuppressive TME and improve the efficacy of immunotherapy and targeted therapy. The two clustering strategies in our study are complementary rather than highly concordant. m6A regulator-based mRGcluster delineates intrinsic epigenetic molecular subtypes of LUAD, whereas RiskScore stratification enables refined prognostic evaluation, immune landscape characterization, genomic mutation profiling and therapeutic response prediction. The former provides epigenetic subtyping background, while the latter offers a quantitative tool for clinical risk stratification, together facilitating multi-level molecular classification and individualized clinical assessment for LUAD patients. Notably, the signature genes were derived from differentially expressed genes between m6A-defined subtypes rather than restricted to canonical m6A regulators. These genes represent downstream transcriptional cascades triggered by m6A epigenetic dysregulation, and thus adequately reflect the biological and prognostic consequences of aberrant m6A modification in LUAD.

Functional enrichment analyses further unravel the molecular underpinnings of mRGcluster-mediated heterogeneity, linking aberrant m6A modification to core malignant phenotypes. The pronounced enrichment of cell cycle progression and DNA replication pathways in mRGcluster B aligns with its poor prognostic profile, as unrestrained cell cycle progression is a hallmark of aggressive tumors. This is mechanistically supported by studies showing m6A regulators like METTL16 drive LUAD cell proliferation by modifying GTSE1 mRNA to stabilize its expression, thereby inhibiting the p53 pathway 17. Consistent with this, enrichment of the p53 signaling pathway in differential genes reinforces that m6A dysregulation disrupts critical cell cycle checkpoints 18. GO analysis detailing mitotic processes (e.g., sister chromatid segregation) and cellular components (e.g., spindle, kinetochore) further contextualizes this, as these structural elements are essential for sustained cell division—likely orchestrated by m6A-mediated stabilization of genes encoding cell cycle regulators 18. Activation of the PI3K-Akt pathway in mRGcluster B adds another layer of significance, as METTL3 has been shown to negatively regulate FGF2 via m6A modification to inhibit PI3K-Akt-mTOR signaling 19. Parallel activation of MYC_TARGETS and E2F_TARGETS further amplifies this pro-tumorigenic circuitry, mirroring findings that METTL14-mediated m6A modification of G6PD drives metabolic reprogramming in LUAD 20. Collectively, these data confirm m6A as an active orchestrator of LUAD heterogeneity with profound implications for targeted therapy 21.

The 8-gene prognostic signature established herein provides a precise tool for LUAD prognosis evaluation and deciphers the molecular network through which m6A dysregulation drives progression. Although multiple m6A-related prognostic signatures have been developed for LUAD, our 8-gene model exhibits distinct incremental strengths. The signature was screened from m6A-associated differentially expressed genes and rigorously validated across multiple independent cohorts, accompanied by multi-dimensional integrative analyses including tumor mutation burden, immune infiltration, therapeutic vulnerability prediction, and single-cell expression verification. Importantly, the RiskScore retained stable independent prognostic performance even in the simplified nomogram without *MKI67*, conferring better clinical interpretability and practical applicability than most existing m6A signatures. Integrating 5 risk factors (ANLN, IGF2BP1, CDKN3, SERPINB5, TMPRSS11E) and 3 protective factors (IRX5, CLIC6, CRTAC1), this signature aligns with prior clinical/mechanistic evidence: ANLN, a core mitotic regulator, enhances LUAD cell proliferation and metastasis via spindle assembly dysregulation 22,23; IGF2BP1, an m6A reader, stabilizes MYC mRNA to promote cancer cell migration 24,25; CRTAC1, a protective factor, inhibits invasion by downregulating MMP2 26. The inclusion of IGF2BP1 reinforces our central model: m6A dysregulation orchestrates LUAD heterogeneity by modulating these core genes. Clinically, this signature could guide personalized therapy—for example, targeting ANLN with spindle poison chemotherapeutics (e.g., paclitaxel) in HR patients 27,28, or inhibiting IGF2BP1 with small-molecule blockers 24.

Hallmark gene set analysis revealed activation of immune checkpoints, EMT, and IL-6/JAK/STAT3 signaling in the HR group, pathways known to facilitate immune evasion 29,30. Elevated activated CD4⁺ T cells, Tregs, and NKT cells in HR patients may contribute to this evasion: Tregs suppress anti-tumor immunity via IL-2 exhaustion 31, 32, while NKT cells weaken immunosurveillance by inhibiting Type I iNKT cells 33. These findings align with immune infiltration data showing RiskScore correlates positively with activated CD4⁺ T cells and negatively with mast cells (Figure 5E), highlighting the signature's utility in predicting TME polarization.

TMB and *CSMD3* mutation analyses further complemented the prognostic implication of RiskScore. The HR group exhibited higher TMB levels (Figure 7D), which generally reflects increased genomic instability 34,35. Although *CSMD3* mutation exerted no significant effect on survival in the overall cohort, it was correlated with improved prognosis specifically within the high-risk subgroup (HRM vs. HRW, *P* < 0.05; Figure 7G). Immune infiltration analysis revealed marginally increased abundances of activated NK cells and follicular helper T cells in HRM patients (Figure 7H). Of note, this subtle immune alteration was restricted to the high-risk subgroup. Considering the intrinsic immune heterogeneity of LUAD and context-dependent functional duality of NK cells within the tumor microenvironment, this unique subgroup-specific immune correlation should be interpreted with caution. Currently, direct biological evidence connecting CSMD3 mutation to the infiltration of these immune subsets remains absent; thus, the observed correlation in this study should be strictly considered an exploratory association rather than a definitive causal relationship.

The RiskScore-based nomogram demonstrates superior prognostic accuracy over traditional indicators (Ki67, clinical stage), with AUC values ranging from 0.70 to 0.84 across cohorts. Although *MKI67* was incorporated into the primary nomogram to improve prognostic performance, it is not routinely standardized in daily LUAD clinical management. We therefore established a simplified nomogram excluding *MKI67* (Supplementary Figure S6) that relies solely on conventional clinical variables combined with RiskScore, which better conforms to real-world clinical application while preserving the stable prognostic value of RiskScore. Bioinformatic predictions based on TIDE, IPS and oncoPredict further revealed subgroup-specific differential therapeutic vulnerability, suggesting that HR patients may have theoretical sensitivity tendency to certain chemotherapeutic agents while LR patients may possess potential phenotypic advantages for immune checkpoint inhibitor response prediction. Notably, these results are derived solely from computational algorithm prediction rather than direct clinical or experimental therapeutic evidence, which should be regarded as hypothesis-generating findings rather than definitive clinical treatment guidance. Unlike conventional biomarkers such as PD-L1 and TMB that focus on single-modality therapeutic prediction 36, our RiskScore integrates prognostic stratification and multi-drug therapeutic vulnerability inference, offering preliminary bioinformatic clues to assist individualized clinical decision reference; nevertheless, prospective clinical trials and functional experiments are still indispensable to validate its practical therapeutic predictive value in real-world LUAD clinical practice.

This study has several limitations. First, the prognostic model is based on retrospective RNA-seq datasets with limited sample sizes, and lacks validation in large independent cohorts and prospective clinical trials to confirm its robustness. In addition, RNA-seq-based modeling may restrict direct clinical applicability due to technical complexity, potential batch effects and higher costs relative to conventional diagnostic assays. Second, the mechanistic basis of the 8-gene signature in regulating m6A modification and tumor immune microenvironment remains underexplored, without sufficient in vitro and in vivo functional experiments to clarify downstream pathways and molecular interactions in LUAD. Third, the cell-cell communication analysis is subject to compositional bias between normal and tumor tissues. The two groups differ substantially in cell-type composition, especially the abundance of malignant epithelial and alveolar epithelial cells; thus, altered interaction number and weight may partially reflect shifts in cellular proportion rather than genuine rewiring of intercellular signaling crosstalk. Accordingly, the interpretation of cell communication changes should be made cautiously, and further experimental verification is needed.

## Conclusion

This study establishes an m6A-related prognostic signature for LUAD, integrating transcriptional, mutational, and immune features to predict survival and therapeutic responses. By bridging epigenetic regulation with clinical outcomes, it provides a basis for personalized risk stratification and treatment selection in LUAD.

## Supplementary Material

Supplementary figures.

Supplementary tables.

## Data Availability

The data presented in this study are publicly accessible through the UCSC Xena portal at https://xenabrowser.net/ (reference number TCGA-LUAD) and the Gene Expression Omnibus at https://www.ncbi.nlm.nih.gov/geo/ (reference numbers GSE41271, GSE42127 and GSE149655). m6A-related genes were identified through the GeneCards database (https://www.genecards.org/) by searching with the keyword 'm6A'. Hallmark gene sets can be obtained from the MSigDB database (https://www.gsea-msigdb.org/gsea/msigdb/human/collections.jsp#H). The TIDE score was obtained by uploading normalized expression data to the TIDE website (http://tide.dfci.harvard.edu/), and IPS can be directly obtained from the LUAD project on the TCIA website (https://tcia.at/home). The training set data (GDSC1 dataset) required for predicting IC50 was downloaded from https://osf.io/c6tfx/overview.

## Abbreviations

LUAD: Lung adenocarcinoma
m6A: N6-methyladenosine
TME: tumor microenvironment
MRGs: m6A-related genes
TMB: tumor mutational burden
mRGclusters: m6A-related gene clusters
OS: overall survival
GEO: Gene Expression Omnibus
KM: Kaplan-Meier
ssGSEA: Single-sample Gene Set Enrichment Analysis
GSEA: Gene Set Enrichment Analysis
DEGs: Differentially expressed genes
HR: high-risk
LR: low-risk
ROC: receiver operating characteristic
TIDE: Tumor Immune Dysfunction and Exclusion
IPS: Immunophenoscore
IC50: half-maximal inhibitory concentration
GDSC: Genomics of Drug Sensitivity in Cancer
GO: Gene Ontology
KEGG: Kyoto Encyclopedia of Genes and Genomes
DO: Disease Ontology
BP: biological processes
CC: cellular components
MF: molecular functions
AUC: area under the curve
EMT: epithelial-mesenchymal transition
NK: natural killer
LRM: LR with mutation
LRW: LR wild-type
HRM: HR mutated
HRW: HR wild-type
