# Supplementary material for: Development and Validation of an m6A-Derived Prognostic Signature in Lung Adenocarcinoma
Source: J Cancer. 2026 May 29;17(6):1134–54. doi: 10.7150/jca.134792 (PMC13280634; doi:10.7150/jca.134792)
Supplement: Supplementary file 1 — Supplementary figures. [file jcav17p1134s1.pdf]

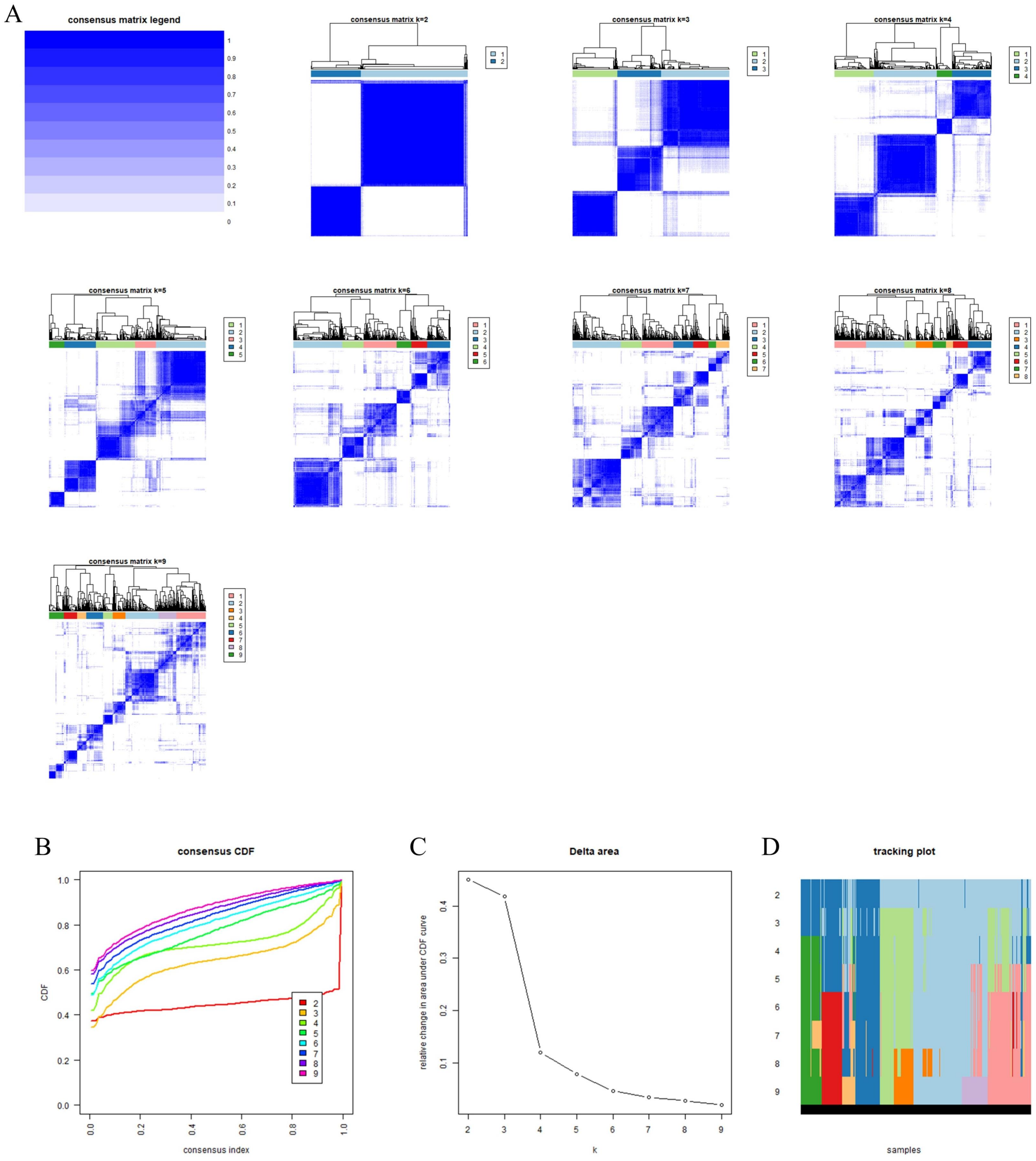

Supplementary Figure S1. Unsupervised clustering of LUAD samples. (A) Unsupervised consensus clustering matrix when k = 1 - 9. (B) Consensus CDF curves showing minimal fluctuations at different consensus indexes. (C) Selection of the optimal clustering number. (D) Trace plot illustrating the clustering of each sample when k values were set from 2 to 9.

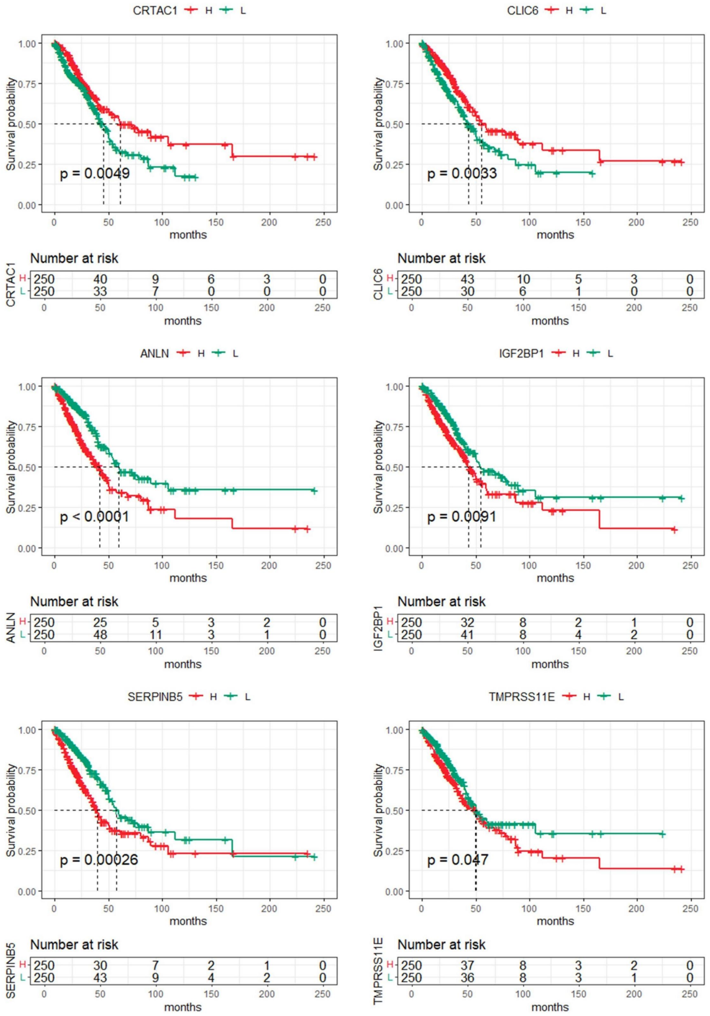

Supplementary Figure S2. KM survival curves for each individual signature gene, comparing OS between high and low expression groups.

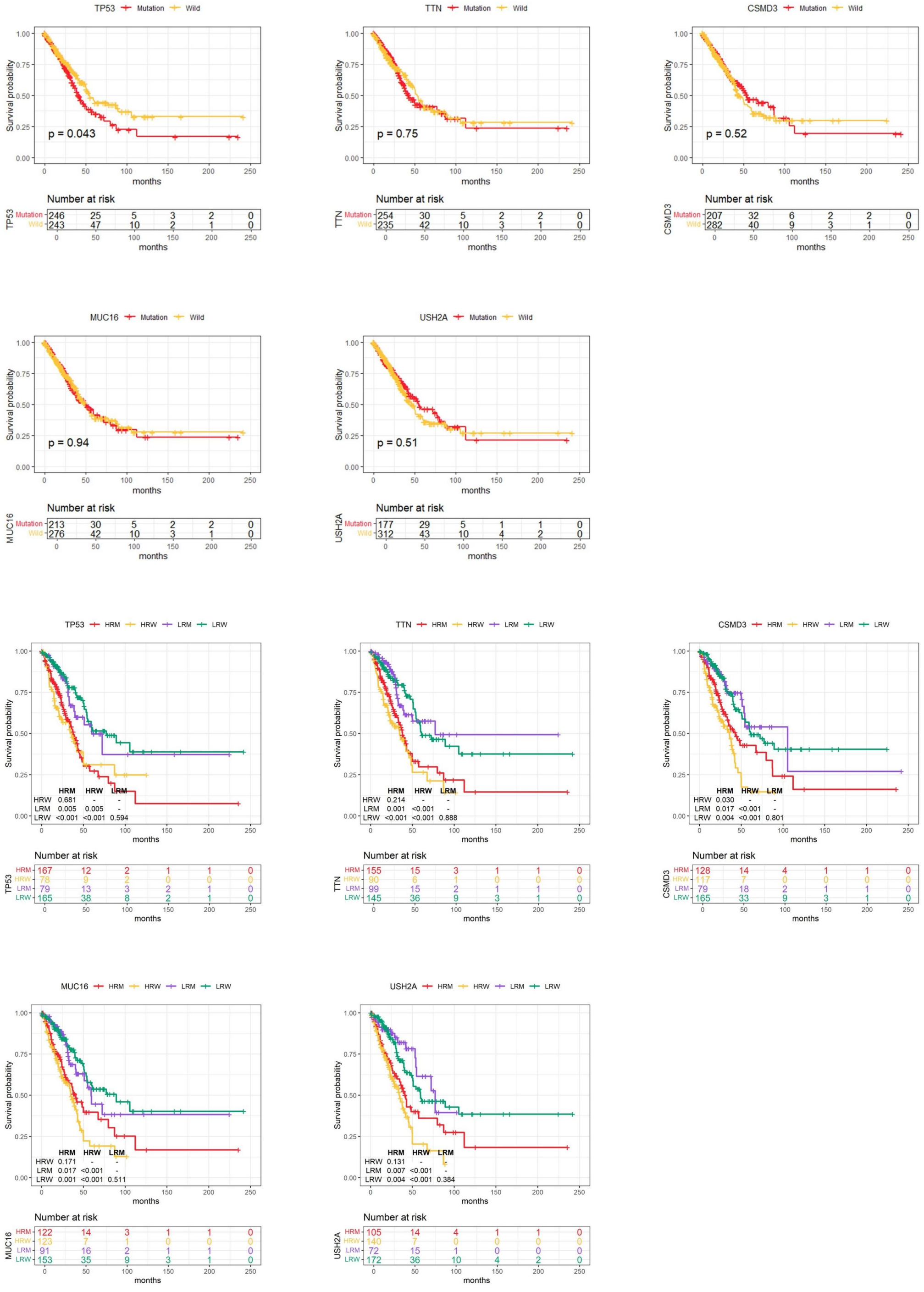

Supplementary Figure S3. Survival analyses of top mutated genes stratified by mutation status and risk subgroups. Kaplan-Meier curves for overall survival comparing mutation vs. wild-type status and four risk-mutation subgroups (HRM, HRW, LRM, LRW) for the top five mutated genes (TP53, TTN, CSMD3, MUC16, USH2A). Log-rank test *P*-values and number-at-risk tables are provided for each comparison.

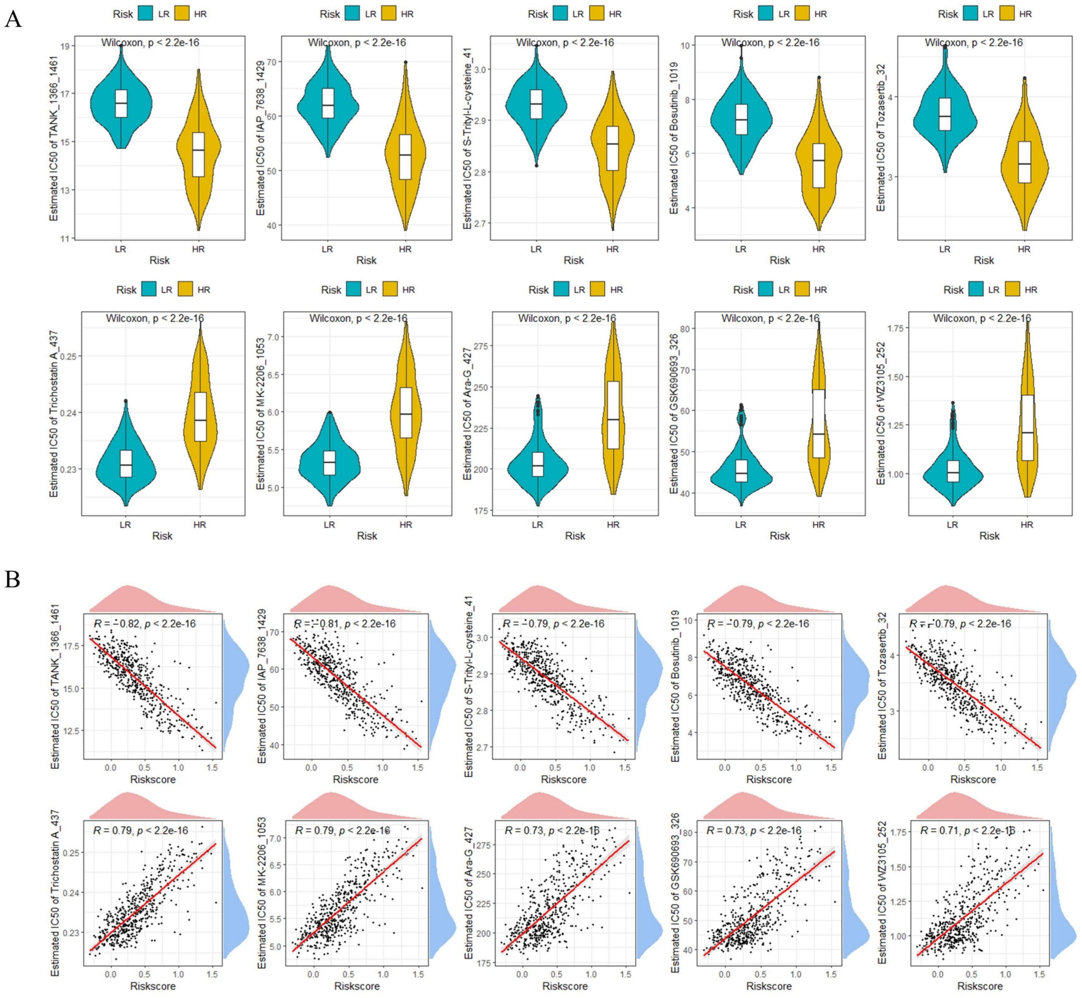

Supplementary Figure S4. (A) Violin plots demonstrating significant IC50 differences of top 5 correlated drugs between LR and HR groups. (B) Scatter plots verifying strong correlations (|r| > 0.7, P < 0.001) between drug IC50 and RiskScore for top 5 correlated agents. **P*< 0.05; ***P*< 0.01; ****P*< 0.001; ns, not significant.

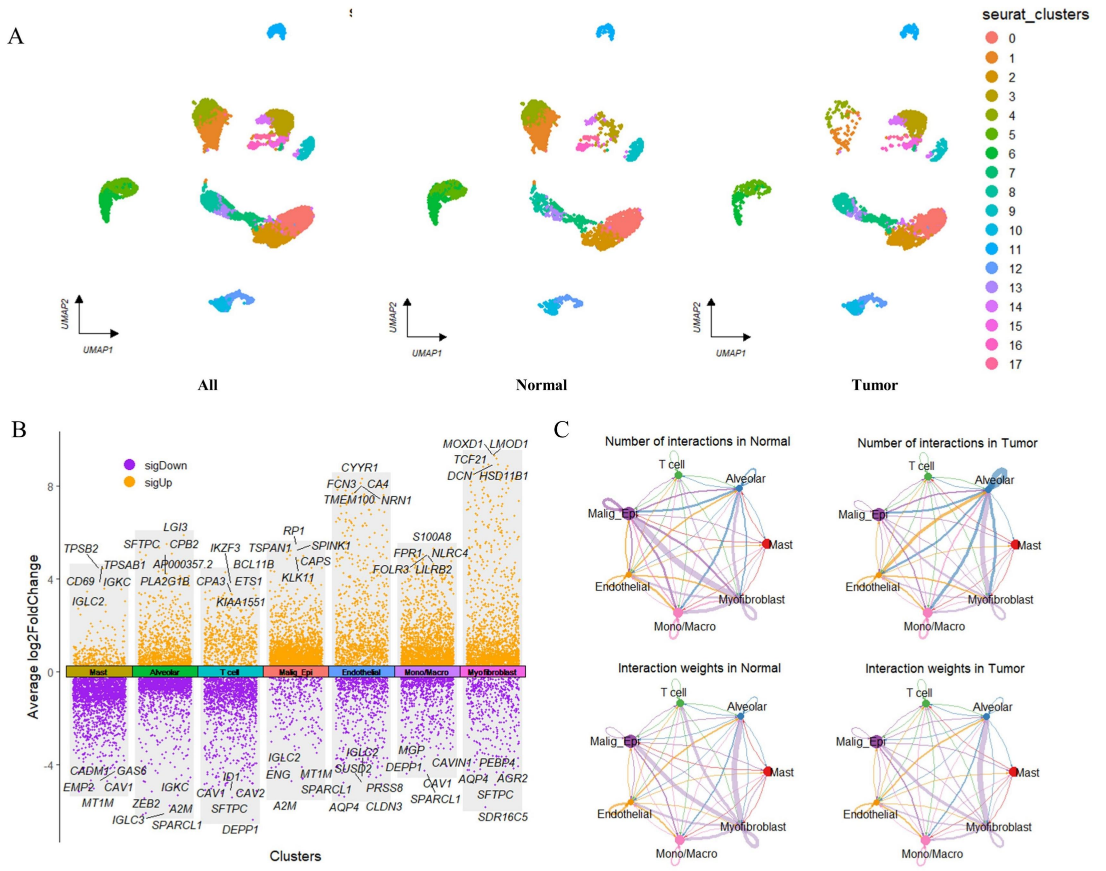

Supplementary Figure S5. Single-cell RNA Sequencing Analysis. (A) UMAP visualization of Seurat-defined clusters in single-cell RNA sequencing data, depicting 18 distinct clusters. (B) Volcano plot of differentially expressed genes across cell clusters, distinguishing significantly upregulated (sigUp) and downregulated (sigDown) genes. (C) Cell-cell interaction networks depicting differences in the number of interactions and interaction weights between normal and tumor tissues.

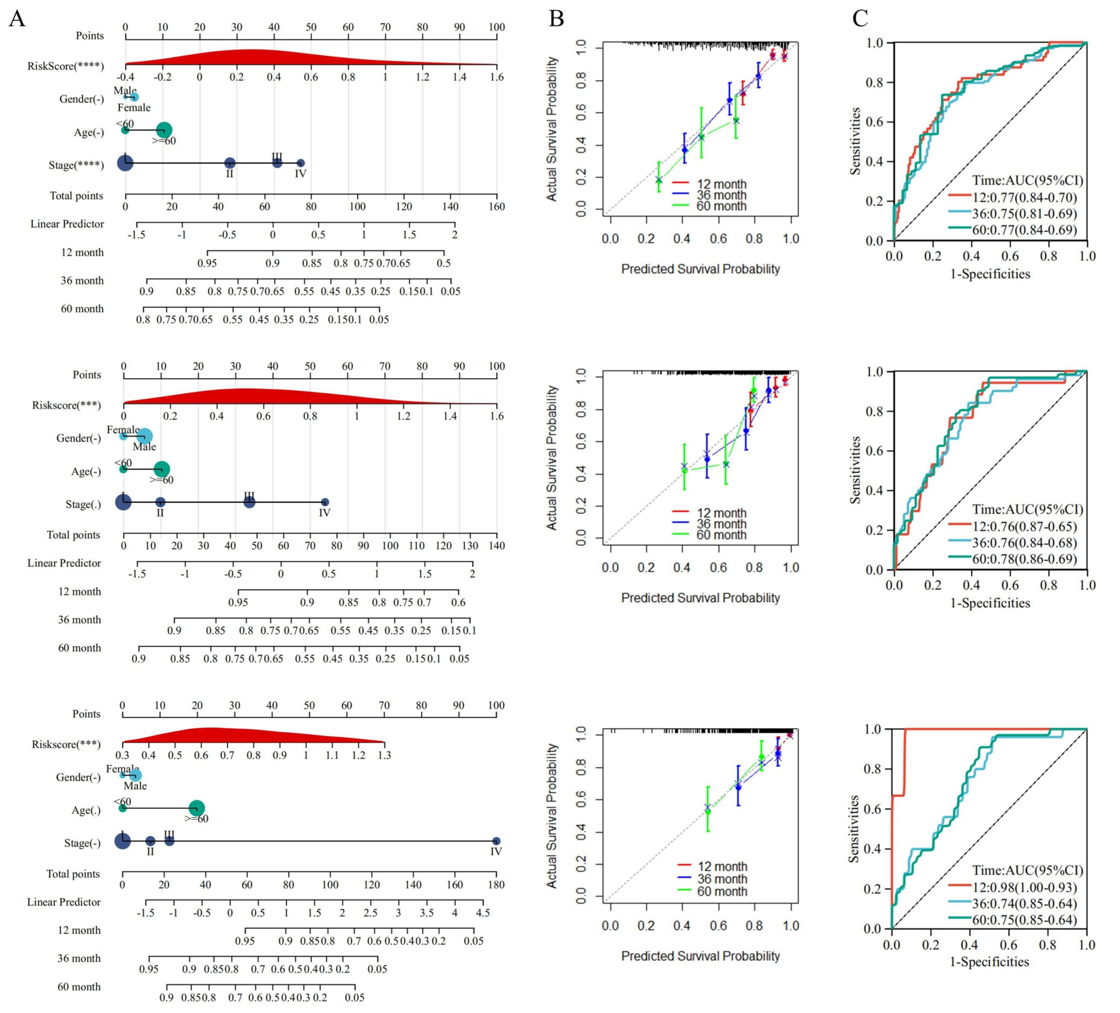

Supplementary Figure S6. RiskScore-Based Prognostic Nomogram for LUAD: Calibration and Discrimination Across Cohorts. (A) Nomograms integrating RiskScore, clinical stage, age, and gender to predict 12-, 36-, and 60-month survival probabilities for TCGA-LUAD, GSE41271, and GSE42127 datasets. (B) Calibration curves comparing predicted and actual survival probabilities at 12, 36, and 60 months across datasets. (C) Time-dependent ROC curves evaluating the model's predictive accuracy for 12-, 36-, and 60-month overall survival. *$P < 0.05$; **$P < 0.01$; ***$P < 0.001$; ns, not significant.
